# Prognostic Value of Blood Ammonia, Lactate, and Brain Natriuretic Peptide in Pediatric Patients With Sepsis: A Prospective, Observational Study

**DOI:** 10.7759/cureus.117450

**Published:** 2026-10-04

**Authors:** Dharmpal Kumar, Amit Kumar, Rakesh Kumar, Jayant Prakash

**Affiliations:** 1 Pediatric Cardiology, Indira Gandhi Institute of Medical Sciences, Patna, IND; 2 Pediatrics, Indira Gandhi Institute of Medical Sciences, Patna, IND

**Keywords:** blood ammonia, brain natriuretic peptide, mortality prediction, pediatric intensive care unit, pediatric sepsis, prognostic biomarkers, prospective cohort study, risk stratification, sepsis outcomes, serum lactate

## Abstract

Background: Early identification of children with sepsis who are at risk of adverse outcomes is essential for timely intervention and optimal management.

Objectives: To evaluate the prognostic significance of serum lactate, blood ammonia, and brain natriuretic peptide (BNP) levels in children with sepsis admitted to the Pediatric Intensive Care Unit (PICU).

Methods: This prospective cohort observational study was conducted in the PICU of the Department of Paediatrics at the Indira Gandhi Institute of Medical Sciences, Patna, over 18 months. Seventy-nine children aged 1 month to 14 years with sepsis were enrolled. Serial measurements of serum lactate and blood ammonia were obtained on day 0, day 3, and day 7, while BNP levels were measured on day 0 and day 3. Clinical outcomes were recorded, and prognostic accuracy was assessed with receiver operating characteristic curve analysis.

Results: Of the 73 patients included in the final analysis, 22 (30.1%) died. Non-survivors demonstrated higher levels of lactate, ammonia, and BNP than survivors. The area under the receiver operating characteristic curve (AUROC) for admission lactate was 0.741, with a cutoff of >3.29 mmol/L, a sensitivity of 54.55%, and a specificity of 92.16%. Ammonia demonstrated an AUROC of 0.713, while BNP had the highest AUROC (0.933) for predicting in-hospital mortality.

Conclusion: Lactate, ammonia, and BNP demonstrated prognostic associations with in-hospital mortality in children with sepsis. BNP showed the highest AUROC among the evaluated biomarkers. These findings should be interpreted cautiously, given the observational design and limited sample size, and should be validated in larger, multicenter cohorts.

## Introduction

Sepsis is a life-threatening condition characterized by organ dysfunction resulting from a dysregulated host response to infection. It remains a leading cause of morbidity and mortality among pediatric patients worldwide [1]. Children, particularly neonates and infants, are highly vulnerable due to immature immune responses, poor physiological reserves, and increased exposure to infectious agents [2]. In low- and middle-income countries, the burden of pediatric sepsis is disproportionately high, with mortality rates ranging from 15% to 40%, especially among those admitted to intensive care units [3]. In India, sepsis constitutes a major proportion of the Pediatric Intensive Care Unit (PICU) admissions, contributing significantly to preventable childhood deaths [4].

Early identification of high-risk patients and timely initiation of aggressive management remain central to improving outcomes in pediatric sepsis. Clinical scoring systems such as the Systemic Inflammatory Response Syndrome (SIRS), the Sequential Organ Failure Assessment (SOFA), and age-adjusted quick SOFA (qSOFA) have been developed to assist in early recognition and prognostication [5]. However, these clinical tools alone may not reliably predict disease severity or mortality in children due to age-related physiological differences and compensated shock states commonly seen in pediatric patients [6]. Hence, there is growing interest in incorporating biochemical markers that reflect underlying organ dysfunction and tissue hypoperfusion to enhance prognostic accuracy.

Blood lactate is widely recognized as a marker of impaired tissue oxygen utilization and cellular hypoxia. Elevated lactate levels have been consistently associated with increased mortality and prolonged PICU stay in septic children [7]. Persistent hyperlactatemia and poor lactate clearance after resuscitation are strong predictors of adverse outcomes [8]. Similarly, hyperammonemia has emerged as a potential indicator of hepatic dysfunction, metabolic stress, and sepsis-associated encephalopathy. Elevated ammonia levels may result from impaired hepatic clearance, increased intestinal absorption, and enhanced protein catabolism during systemic inflammation and have been linked to higher mortality in critically ill pediatric patients [9,10].

Brain natriuretic peptide (BNP), a hormone released in response to myocardial stretch and inflammation, has gained importance as a marker of sepsis-induced myocardial dysfunction. Several studies have demonstrated that elevated BNP and NT-proBNP levels correlate with disease severity, need for inotropic support, and mortality in pediatric septic shock [11,12]. As cardiovascular dysfunction plays a pivotal role in sepsis-related organ failure, BNP may serve as a valuable prognostic biomarker.

Integrating biochemical markers with clinical scoring systems such as qSOFA may provide a more comprehensive assessment of disease severity and prognosis. In this context, the present study, conducted at the PICU of the Department of Paediatrics, Indira Gandhi Institute of Medical Sciences, Patna, aims to evaluate the prognostic value of blood ammonia, lactate, and BNP levels in pediatric patients with sepsis. By identifying reliable predictors of adverse outcomes, this article seeks to enhance early risk stratification, guide timely interventions, and ultimately improve survival in critically ill children, in alignment with global recommendations such as those proposed by the Surviving Sepsis Campaign.

## Materials and methods

Study design and setting

This prospective observational cohort study was conducted at the PICU of the Department of Paediatrics, Indira Gandhi Institute of Medical Sciences, Patna, India, over a period of 18 months after obtaining approval from the Institutional Ethics Committee. The study aimed to evaluate the prognostic significance of blood ammonia, serum lactate, and BNP levels in predicting clinical outcomes among pediatric patients with sepsis admitted to the PICU.

Study population, definition, and clinical classification of sepsis

Children aged 1 month to 14 years admitted to the PICU with suspected or confirmed infection were considered eligible for enrollment. Patients were recruited over an 18-month period from April 2023 to October 2024. For the present study, sepsis was defined as a suspected or confirmed infection accompanied by fulfillment of the SIRS criteria and/or an age-adjusted qSOFA score of ≥2. Patients were clinically classified by illness severity documented during hospitalization, including sepsis, severe sepsis, and septic shock, where applicable. The criteria used for patient classification were based on the clinical findings, organ dysfunction, and hemodynamic status documented at admission and during the PICU stay. These criteria were applied consistently for patient identification and enrollment throughout the study period. The SIRS-based pediatric criteria were used because they were the criteria specified in the study protocol during the recruitment period; the authors acknowledge that pediatric sepsis definitions have subsequently evolved, with the 2024 international consensus criteria recommending the Phoenix Sepsis Score for identification of pediatric sepsis. Written informed consent was obtained from parents or legal guardians before enrollment.

Inclusion and exclusion criteria

Children aged between 1 month and 14 years admitted to the PICU with suspected or confirmed sepsis who fulfilled the SIRS criteria and/or had an age-adjusted qSOFA score of ≥2 were eligible for inclusion. Only patients whose parents or legal guardians provided written informed consent and who underwent serial biomarker assessment according to the study protocol were included in the final analysis.

Patients with known inborn errors of metabolism, chronic liver disease or hepatic failure, underlying malignancy, chronic renal failure requiring dialysis, congenital or acquired cardiac disease likely to influence BNP concentrations, significant gastrointestinal bleeding, or metabolic disorders associated with hyperammonemia were excluded, as these conditions could independently alter the study biomarkers. Patients discharged against medical advice or with incomplete clinical outcome data were excluded from the final analysis. Patients were not excluded solely because day 3 or day 7 biomarker measurements were unavailable due to death or discharge before the scheduled sampling time.

Sample size calculation

The sample size was estimated using the single population-proportion formula:

n = Z² × p (1 − p) / e²

where n represents the required sample size, Z is the standard normal deviate corresponding to a 95% confidence level (1.96), p is the estimated prevalence of pediatric sepsis (25%), and e is the allowable margin of error (10%). Based on these assumptions, the minimum required sample size was calculated to be 72 participants. After accounting for an anticipated attrition rate of approximately 10%, the target sample size was increased to 79 participants. Because the primary objective of the study was to evaluate the prognostic performance of biomarkers for in-hospital mortality, rather than to estimate the prevalence of pediatric sepsis, this prevalence-based sample size calculation was not specifically powered for prognostic discrimination or area under the receiver operating characteristic curve (AUROC) estimation.

Study procedure

Following enrollment, all patients underwent detailed clinical evaluation at admission. Demographic characteristics, clinical findings, vital signs, qSOFA scores, microbiological investigations, hematological parameters, and biochemical investigations were recorded using a predesigned case record form. The presence of organ dysfunction and hemodynamic instability was documented during the PICU stay to characterize the clinical severity of sepsis, including severe sepsis and septic shock where applicable. All enrolled patients received standard management for pediatric sepsis according to institutional PICU protocols. Serial measurements of blood ammonia, serum lactate, and BNP were obtained according to the predefined study schedule, and patients were monitored prospectively until discharge from the hospital or death.

Biomarker measurement protocol

Blood samples were collected under strict aseptic precautions at predefined time points during hospitalization. Serum lactate and blood ammonia were measured on day 0 (within six hours of PICU admission), day 3, and day 7. BNP levels were measured on day 0 and day 3 because of financial constraints and limited assay availability. Patients who died or were discharged before a scheduled day 3 or day 7 measurement were not available for subsequent sampling. Such measurements were considered unavailable; no values were imputed, and patients were not excluded from the overall outcome analysis solely because of missing subsequent biomarker measurements.

For serum lactate estimation, arterial blood samples were collected in heparinized syringes and analyzed immediately using the arterial blood gas (ABG) analyzer available in the PICU. The analyzer underwent routine internal quality-control procedures according to the manufacturer's recommendations, and prompt analysis minimized pre-analytical variation.

Blood ammonia estimation was performed using venous blood collected in ethylenediaminetetraacetic acid tubes. Samples were transported on ice and analyzed immediately using a point-of-care ammonia analyzer based on a single-wavelength reflectance method to avoid falsely elevated values caused by delayed processing.

Serum BNP concentrations were measured from venous blood samples collected in plain tubes. After centrifugation, serum samples were analyzed in the central laboratory using a chemiluminescent microparticle immunoassay (CMIA) according to the manufacturer's instructions. Routine internal quality-control procedures were followed, and laboratory reference ranges were used for interpretation.

Outcome measures

The primary outcome of the study was in-hospital mortality, defined as death occurring during the same hospital admission following PICU admission for sepsis.

Secondary outcomes included the length of PICU stay (total days from admission until discharge or death), survival status at hospital discharge, and the prognostic performance of serial blood ammonia, serum lactate, and BNP measurements in predicting mortality among children with sepsis.

Statistical analysis

Data were entered into MS Excel (Microsoft Corporation, Redmond, Washington) and analyzed using IBM SPSS Statistics for Windows, Version 23 (Released 2025; IBM Corp., Armonk, New York). Continuous variables were tested for normality using the Shapiro-Wilk test. Normally distributed variables were expressed as mean ± standard deviation (SD), whereas non-normally distributed variables were summarized as the median with interquartile range (IQR). Comparisons between survivors and non-survivors at individual time points were performed using the independent Student's t-test for normally distributed continuous variables and the Mann-Whitney U test for non-normally distributed variables. Categorical variables were presented as frequencies and percentages and compared using the chi-square test or Fisher's exact test, as appropriate.

For serial biomarker measurements obtained from the same patients at multiple time points, repeated-measures analysis was performed to account for within-patient correlation. A linear mixed-effects model was used to assess changes in biomarker levels over time and differences in biomarker trajectories between survivors and non-survivors, with patient-level measurements treated as repeated observations. The interaction between time and outcome group was evaluated to determine whether biomarker trajectories differed between survivors and non-survivors.

Receiver operating characteristic (ROC) curve analysis was performed to evaluate the prognostic performance of serum lactate, blood ammonia, and BNP levels for predicting in-hospital mortality. The AUROC with 95% confidence intervals was calculated for each biomarker. Optimal cutoff values were determined using the Youden index, and sensitivity, specificity, positive predictive value (PPV), and negative predictive value (NPV) were calculated. A two-sided P value of less than 0.05 was considered statistically significant.

Handling of missing serial biomarker measurements

Serial biomarker analyses were performed using the measurements available at each scheduled time point. Missing biomarker values were not imputed. The number of available lactate and ammonia measurements at day 0, day 3, and day 7 was reported separately for survivors and non-survivors. The potential effect of differential availability of serial measurements on longitudinal biomarker comparisons was considered when interpreting the findings.

## Results

A total of 79 children meeting the eligibility criteria were enrolled during the study period. Six patients were excluded from the final analysis because of incomplete serial biomarker measurements or missing outcome data, leaving 73 patients for the final analysis.

Demographic profile

The age-wise distribution of patients is presented in Table 1. Infants aged below one year constituted the largest group (32.9%), followed by children aged 1-5 years (30.1%). Collectively, 63% of the study population was below five years of age, indicating that younger children were more frequently affected by severe sepsis requiring intensive care. Children aged 6-10 years accounted for 26%, while those older than 10 years formed the smallest subgroup (11%). It also presents the demographic characteristics of the study population.

**Table 1:** Age-wise distribution of study participants (n = 73).

Age group (years)	Number	Percentage (%)
<1	24	32.9%
1-5	22	30.1%
6-10	19	26.0%
>10	8	11.0%
Total	73	100%

Gender distribution (Table 2) revealed a higher proportion of male patients (63%) than female patients (37%), suggesting a male predominance among septic admissions to the PICU.

**Table 2:** Gender distribution.

Gender	Number	Percentage (%)
Male	46	63%
Female	27	37%
Total	73	100%

Clinical outcomes and hospital stay

The outcome analysis shown in Table 3 demonstrated that 51 children (69.9%) survived, whereas 22 patients (30.1%) succumbed to the illness. This reflects a substantial mortality burden associated with pediatric sepsis in the intensive care setting.

**Table 3:** Clinical outcome of patients.

Outcome	Number	Percentage (%)
Survived	51	69.9%
Expired	22	30.1%
Total	73	100%

The duration of the PICU stay is summarized in Table 4. The mean length of stay was 14.81 ± 6.01 days, with a median of 15 days and a wide range from 1 to 29 days. This indicates prolonged intensive care requirements among affected children, reflecting disease severity and complications during treatment.

**Table 4:** Duration of Pediatric Intensive Care Unit (PICU) stay. IQR: interquartile range.

Parameter	Value
Mean ± SD (days)	14.81 ± 6.01
Median (IQR)	15 (10-19.5)
Range	1-29

Microbiological findings

Culture results are detailed in Table 5. *Staphylococcus* species were the most frequently isolated pathogens, accounting for 29.2% of cases. This was followed by *Klebsiella* species (23.1%), *Escherichia coli* (9.2%), and *Pseudomonas* species (7.7%). Fungal infections due to *Candida* species were identified in 7.7% of cases. Other organisms comprised 23.1% of isolates. These findings highlight a mixed microbiological profile, with a predominance of gram-positive organisms; *Staphylococcus* species were the most frequently isolated (29.2%), followed by *Klebsiella* species (23.1%). The composition of the “Others” category was not further subdivided in the present analysis.

**Table 5:** Culture-positive organisms isolated.

Organism	Number	Percentage (%)
*Staphylococcus* species	19	29.2%
*Klebsiella* species	15	23.1%
Escherichia coli	6	9.2%
*Pseudomonas* species	5	7.7%
*Candida* species	5	7.7%
Others	15	23.1%

Comparison of laboratory parameters

Table 6 compares hematological and biochemical variables between survivors and non-survivors. Non-survivors had significantly higher neutrophil percentages (77.59 ± 11.63) than survivors (69.63 ± 16.07), while lymphocyte counts were markedly lower among non-survivors (14.18 ± 9.87 vs 23.92 ± 15.11). Platelet counts were significantly reduced in the non-survivor group, indicating coagulopathy and systemic inflammation.

**Table 6:** Comparison of laboratory parameters between survivors and non-survivors. SGOT: serum glutamic-oxaloacetic transaminase; PT: prothrombin time.

Parameter	Survivors (mean ± SD)	Non-survivors (mean ± SD)	P value
Neutrophils (%)	69.63 ± 16.07	77.59 ± 11.63	0.04
Lymphocytes (%)	23.92 ± 15.11	14.18 ± 9.87	<0.01
Platelets (×10^9^/L)	281.45 ± 181.26	197.45 ± 21.96	0.02
SGOT (U/L)	178.35 ± 351.72	215.27 ± 242.80	0.01
PT-seconds	20.80 ± 7.66	28.33 ± 16.91	<0.01
D-dimer (mg/dL)	1.61 ± 0.53	2.33 ± 1.41	<0.001

Markers of organ dysfunction were also elevated in non-survivors, with significantly higher serum glutamic-oxaloacetic transaminase (SGOT) levels, prolonged prothrombin time (PT)-seconds, and increased D-dimer values. These findings demonstrate widespread hepatic involvement and coagulation abnormalities in patients with fatal outcomes.

Availability of Serial Biomarker Measurements

At baseline (day 0), lactate and ammonia measurements were available for all 51 survivors and 22 non-survivors. On day 3, lactate measurements were available for 47 survivors (92.2%) and 14 non-survivors (63.6%), while ammonia measurements were available for 46 survivors (90.2%) and 13 non-survivors (59.1%). On day 7, lactate measurements were available for 41 survivors (80.4%) and 7 non-survivors (31.8%), while ammonia measurements were available for 40 survivors (78.4%) and 6 non-survivors (27.3%).

Serial Serum Lactate Levels

Serum lactate trends are illustrated in Table 7. At admission (day 0), lactate levels were significantly higher among non-survivors (4.56 ± 3.44 mmol/L) than among survivors (2.58 ± 0.61 mmol/L). This difference persisted on day 3 and day 7, with non-survivors showing sustained hyperlactatemia, while survivors exhibited progressive decline in lactate levels over time. The differences at all time points were statistically significant.

**Table 7:** Serial serum lactate levels among survivors and non-survivors.

Time point	Survivors (n)	Mean ± SD	Non-survivors (n)	Mean ± SD	P value
Day 0	51	2.58 ± 0.61	22	4.56 ± 3.44	<0.01
Day 3	47	1.91 ± 0.74	14	3.64 ± 2.43	<0.001
Day 7	41	1.02 ± 0.48	7	3.89 ± 2.40	<0.001

Prognostic Value of Lactate

The diagnostic performance of admission lactate is presented in Table 8. A cutoff value greater than 3.29 mmol/L demonstrated a sensitivity of 54.55% and a high specificity of 92.16% for predicting mortality. The PPV was 75%, and the NPV was 82.46%. The AUROC was 0.741, indicating good discriminative ability of lactate as a prognostic marker. The diagnostic performance of blood ammonia at admission is shown in Table 9. Serum BNP levels at day 0 and day 3 in survivors and non-survivors are summarized in Table 10. The diagnostic performance of BNP at admission is reported in Table 11, and the AUROCs of qSOFA, ammonia, lactate, and BNP for predicting mortality are compared in Table 12.

**Table 8:** Ammonia levels: survived vs expired. (units: µmol/L). Survivors showed a declining trend over time; non-survivors stayed persistently elevated.

Time point	Survivors (n)	Mean ± SD	Non-survivors (n)	Mean ± SD	P value
Day 0	51	75.09 ± 9.06	22	83.30 ± 9.69	<0.01
Day 3	46	55.13 ± 9.51	13	86.24 ± 11.53	<0.001
Day 7	40	35.49 ± 10.32	6	88.19 ± 21.51	<0.001

**Table 9:** Diagnostic value of ammonia (day 0) for predicting mortality. AUC = 0.713, cut-off >88.5 µmol/L (good for ruling out mortality - high NPV/sensitivity). AUC: area under the curve; NPV: negative predictive value; PPV: positive predictive value.

Metric	Value	95% CI
Sensitivity	90.00%	68.30-98.77%
Specificity	53.49%	37.65-68.82%
PPV	47.37%	38.76-56.14%
NPV	92.00%	74.99-97.78%
Accuracy	65.08%	52.03-76.66%
+LR	1.93%	1.36-2.75%
-LR	0.19%	0.05-0.72%

**Table 10:** Brain natriuretic peptide (BNP) levels: survived vs expired. (units: pg/mL; note BNP was measured only at day 0 and day 3, not day 7, unlike lactate/ammonia). BNP: brain natriuretic peptide.

Day	Survived (mean ± SD)	Expired (mean ± SD)
Day 0	612.02 ± 164.42	926.91 ± 141.61
Day 3	392.69 ± 131.32	848.42 ± 121.42

**Table 11:** Diagnostic value of BNP (day 0) for predicting mortality. AUC = 0.933, cut-off >773 pg/mL, the best single predictor of the three biomarkers. BNP: brain natriuretic peptide; PPV: positive predictive value; NPV: negative predictive value.

Metric	Value	95% CI
Sensitivity	81.82%	59.72-94.81%
Specificity	94.12%	83.76-98.77%
PPV	85.71%	66.29-94.82%
NPV	92.31%	83.14-96.69%
Accuracy	90.41%	81.24-96.06%
+LR	13.91%	4.56-42.43%
-LR	0.19%	0.08-0.47%

**Table 12:** BNP demonstrated the highest area under the receiver operating characteristic curve (AUROC) numerically among the evaluated biomarkers. BNP: brain natriuretic peptide; qSOFA: quick Sequential Organ Failure Assessment; AUROC: area under the receiver operating characteristic curve.

Variable	AUC	P value	95% CI
qSOFA	0.640	0.056	0.501-0.787%
Ammonia (day 0)	0.713	0.007	0.578-0.849%
Lactate (day 0)	0.741	0.002	0.593-0.888%
BNP (day 0)	0.933	0.000	0.873-0.994%

## Discussion

This prospective cohort study, conducted at the PICU of Indira Gandhi Institute of Medical Sciences, demonstrates the continued high burden of pediatric sepsis and highlights the potential prognostic significance of biochemical biomarkers, including serum lactate, blood ammonia, and BNP, in predicting mortality. The predominance of younger children in the present cohort reflects the vulnerability of early childhood to severe infection, consistent with previous pediatric intensive care studies [4]. In the present study, the three biomarkers demonstrated different patterns of prognostic performance, with lactate showing significant differences between survivors and non-survivors, while ammonia and BNP provided additional biochemical indicators of disease severity and outcome.

The observed mortality rate of 30.1% remains comparable to rates reported in similar tertiary care settings across developing nations. Regional studies have documented pediatric sepsis mortality ranging between 25% and 40%, despite advances in antimicrobial therapy and critical care support [3]. This emphasizes the need for early risk stratification and aggressive management strategies in resource-limited environments.

A major finding of this study was the strong association between elevated serum lactate levels and adverse outcomes. Non-survivors demonstrated significantly higher lactate concentrations at admission and persistent hyperlactatemia during hospitalization. Previous investigations have shown that poor lactate clearance is associated with impaired tissue perfusion and increased risk of multiorgan failure in septic children [7,13]. Serial lactate monitoring has been advocated as a dynamic indicator of therapeutic response and hemodynamic stabilization, offering superior prognostic value compared to single-point measurements [8,14].

The present study also identified significant hematological and coagulation abnormalities among non-survivors, including thrombocytopenia, prolonged PT-international normalized ratio (INR), and elevated D-dimer levels. These changes reflect sepsis-induced coagulopathy and disseminated intravascular coagulation, which are well-established predictors of mortality in critically ill pediatric populations [15,16]. Similar patterns have been observed in multicenter pediatric cohorts, where early platelet decline and coagulation derangements correlated strongly with organ dysfunction and fatal outcomes [9].

Hepatic dysfunction was evident in non-survivors, as indicated by higher transaminase levels. Liver injury during sepsis results from hypoperfusion, inflammatory mediator release, and mitochondrial dysfunction, contributing to metabolic instability and impaired detoxification. Prior studies have reported a strong association between rising liver enzymes and increased mortality, underscoring the prognostic relevance of hepatic involvement in septic shock [17,18].

The microbiological spectrum in this cohort demonstrated a mixed distribution of bacterial and fungal isolates. *Staphylococcus* species were the most frequently identified individual organism group, followed by *Klebsiella* species, *E. coli*, and *Pseudomonas* species. Because the organisms included in the "Others" category were not individually specified, overall gram-positive vs gram-negative predominance could not be reliably determined. This shift toward gram-positive dominance has been increasingly reported in intensive care units, likely due to invasive devices, prolonged hospital stays, and antibiotic selection pressure [10,19]. Recognition of local pathogen profiles is essential for optimizing empirical antimicrobial therapy and improving outcomes.

Overall, lactate, ammonia, and BNP demonstrated different patterns of prognostic performance in the present cohort. BNP showed the highest AUROC among the evaluated biomarkers, consistent with prior reports describing BNP as a marker of myocardial dysfunction and adverse prognosis in severe sepsis [11,12], while serial lactate levels were consistently higher among non-survivors and demonstrated prognostic discrimination. These findings suggest that all three biomarkers may provide complementary information regarding prognosis in pediatric sepsis. However, because the study was observational and the analyses were unadjusted, the findings should be interpreted as associations rather than independent predictive superiority.

Recent literature further emphasizes the clinical heterogeneity and potential severity of invasive infections in neonatal and pediatric populations, including severe bacterial infections and infections requiring advanced antimicrobial therapy. These reports highlight the importance of early recognition, appropriate assessment of disease severity, and timely antimicrobial management in critically ill children [20,21].

Clinical applicability

The findings of the present study have important clinical implications for the management of pediatric sepsis in intensive care settings. Serial monitoring of serum lactate may serve as a simple, rapid, and objective tool for early risk stratification and prognostication in critically ill children with sepsis. Persistently elevated lactate levels or inadequate lactate clearance during hospitalization may help clinicians identify patients at increased risk of adverse outcomes. This can facilitate timely escalation of treatment, closer hemodynamic monitoring, optimization of antimicrobial therapy, and early referral for advanced organ support when indicated. As serum lactate estimation is widely available, relatively inexpensive, and can be performed rapidly using point-of-care blood gas analyzers, its incorporation into routine sepsis management protocols may support clinical decision-making, particularly in resource-limited settings. Nevertheless, serum lactate should not be interpreted in isolation but rather as an adjunct to comprehensive clinical assessment, established severity scoring systems, and other laboratory parameters to guide individualized patient management.

Limitations

This study has several limitations. First, it was conducted at a single center with a relatively small sample size, which may limit the generalizability and precision of the findings. Second, serial biomarker measurements were not available for all patients because some patients died or were discharged before the scheduled follow-up measurements, which may have introduced potential survivorship or informative missingness bias. The decreasing availability of serial measurements over time, particularly among non-survivors, also limited longitudinal comparisons and may have affected the interpretation of temporal biomarker trends. Third, the study did not include multivariable adjustment for potential confounding factors; therefore, the independent prognostic value of each biomarker could not be established. Fourth, the biomarker cutoff values were derived from the same cohort and were not externally validated, which may result in optimistic estimates of prognostic performance. Fifth, the sample-size calculation was based on disease prevalence rather than biomarker-specific prognostic discrimination and was not specifically powered to detect differences in AUROC or other measures of prognostic performance. Sixth, BNP measurement was restricted by financial and assay constraints. Finally, the study did not include long-term follow-up, and the findings require confirmation in larger, multicenter cohorts with external validation.

## Conclusions

Serum lactate, blood ammonia, and BNP demonstrated prognostic potential in children with sepsis. BNP showed the highest AUROC among the evaluated biomarkers, while serial lactate levels were consistently associated with mortality. These findings support the utility of these biomarkers in prognostic assessment in pediatric sepsis. However, given the observational design, limited sample size, and unadjusted analyses, the findings should be interpreted cautiously and validated in larger, multicenter studies.
